# Foreign Medical Literature

**Published:** 1819-07-01

**Authors:** 


### Second Department.

### FOREIGN MEDICAL LITERATURE.

### I.

### *On Ingurgitation, and a convulsive Species of Ebriety, considered in a Medical, Moral, and Forensic Point of View.*

[Partly from the French of M. Friedlander, Percy, Laurent, &c.]

1. *Drunkards.* The most sober individual may occasionally be drawn into a degree of inebriety, almost unconsciously; but he who *accustoms* himself to the use of strong fermented liquors gradually becomes what may be termed a *drinker*, and, if he check not the propensity, slides insensibly into a—*drunkard!* Few people pass through life without experiencing a fit of ebriety, and the sufferings, in such cases, are only temporary; but the nervous sensibility of the confirmed inebriate is daily blunted, and at length ceases to obey the action of stimulants. While these changes are going on in the body, a corresponding deterioration is manifest in the mind. A train of *evil dispositions* is too often generated, ending ultimately in sottishness. Drunkenness is, therefore, a real disease of the corporeal and mental functions.

When we reflect on the irresistible propensity towards intoxicating drinks which many people evince, we can hardly help attributing it to a morbid germ which has grown up in the constitution, or been transmitted from parent to progeny. The example of the father, indeed, must have a baneful effect on the mind of the child; and this is rendered still worse by the pernicious custom of giving wine, however small the quantity, to children and youths, by which the tone and functions of their digestive organs are deteriorated, and turbulent passions and dispositions engendered or developed. The female sex too, imbibes the contagion, and in them it exerts the most deleterious influence. Witness those unfortunate females who drown the memory of the past, the frightful anticipations of the future—the remains of moral feeling, and the bloom of health, in the ocean of ebriety!

There is an external character, a manner, an aspect in the inebriate, even when sober, which stamps him unequivocally, and distinguishes him from the man of habitual temperance. He becomes heavy and aukward in his gait; bloated in his countenance; his eyes and eye-lids are inflamed; he falters in his speech; his nose is red; his complexion sallow; his face covered with eruptions or excrescences; his abdomen rather tumid; his breath is fœtid. He is subject to nausea in the mornings; his respiration is short, like that of an asthmatic; his stools are morbid in colour and smell; his urine turbid or sedimentous. His skin and muscles are flaccid; his hands tremble. He emaciates, and is overtaken with premature old age!

The manifestations of the soul correspond with the derangements of the corporeal organs and functions. He is incapable of attention; fails in his memory and judgment; becomes irresolute; timid—even cowardly. The morning hours hang heavy upon him, and he is miserable till he gets once more immersed in the fumes of the vinous or spirituous debauch! Finally, he sinks into sottishness and stupidity, and dies paralytic, apoplectic, asthmatic, dropsical, or maniacal.

It is on the digestive organs of the drunkard, however, that the evil effects of his intemperance more particularly fall. The liver and its secretions are deteriorated in a thousand different ways. In the distilleries and breweries, where hogs and fowls are fed on the grains left after distillation and fermentation, we find the livers of these creatures scirrhous, indurated, and enlarged. So in drunkards, the constant irritation in the line of the digestive organs keeps up a determination of blood to those viscera, ending in congestion, chronic inflammation, and obstruction. The urinary organs, too, suffer from calculous affections, especially in the *wine bibber;* but it is a remarkable fact that *beer drinking* rather confers an immunity from stone. Haller opened the bodies of three hundred and fifty beer-drunkards, and only found two instances of calculus. Cyprianus, on the other hand, who is said to have performed the operation of lythotomy fourteen hundred times, states that the greater number of his patients were wine drinkers.

The thoracic organs of the drunkard come in for a share of the consequences of inebriety. Hydrothorax, and structural as well as functional diseases of the heart, are the prominent features in this class, as has been amply shewn by Morgagni and Corvisart, and indeed as may be every day seen in this country. Intemperance in drink, as was before remarked, has a peculiar effect on the skin, in the way of eruptions, probably from the sympathy between the surface of the body and the liver. The rubicund nose of the drunkard may be accounted for in the same way. This feature of ebriety has been noticed both by poets and physicians. Shakspeare, that accurate observer of Nature, has given a striking and grotesque picture of a drunkard's nose, on the face of Bardulph. He compares it to a lighted coal, which serves to guide him from tavern to tavern, instead of a lantern. It is needless to say that all diseases, to which an individual is previously disposed, are exasperated by intemperance in drink; particularly gout and rheumatism, the latter probably from exposure to atmospheric vicissitudes, as is exemplified among sailors and soldiers. The generative system, in both sexes, suffers from inebriety; even Plutarch remarks, that the progeny of drunkards are generally worthless. How much the brain and nervous system are deranged from excessive inebriation our Lunatic Assyla can mournfully witness! Of two hundred and sixty-four female lunatics in the Salpetriere at Paris, twenty-six became insane from intoxication. In the male sex the proportion is still greater; and in countries such as England and Holland, where more spirits and beer are drunk than in France, the number of insane persons from drink far exceeds that which is witnessed in the latter country.

2. *Drunkenness.* Every one knows the pleasing sensations which a few glasses of wine diffuse over mind and body. A sort of Elysium opens round the soul; the world presents nothing but flattering visions of happiness and prosperity; love, friendship, liberality, take possession of the breast; and if this scene could be prolonged, man would have little reason to envy the situation of the Gods. Indeed, Homer generally represents the guests of Jupiter, and even the Sceptre-bearer himself, as passing the bowl freely round the table. But the more exhalted the pleasure, the more bitter the pain; the more vivid the excitement, the more speedy the collapse. Let us visit this jovial assembly a few hours later in the evening, and we shall find———

——— Their feeble tongues,
Unable to take up the cumbrous word,
Lie quite dissolved. Before their maudlin eyes,
Seen dim and blue, the double tapers dance
Like the sun wading through the misty sky.

If we examine the physical phenomena which attend this series of changes, from sobriety to stupor, we shall not be surprised at the serious consequences which must ensue from such a perturbation of the various functions. The action of the heart is inordinately excited, and the blood is driven with great violence to the head in particular, as is evinced by the florid swelling of the face; the turgescence of the vessels of the neck and temples; in short——

Polyphemus ——— in cerebro
Cum suis Cyclopibus malleat!

All the passions are turned loose, and without reason to restrain them :—in fact, the nervous and vascular systems are thrown into the most wild and tumultuous confusion.

After a stertorous sleep, or rather stupor, the inebriate awakes, and finds the Elysian fields transformed into a dreary desert! All the phenomena of arterial excitement are now changed into those attending venous congestion. The action of the heart in truth, is so enfeebled after the orgasm, that this organ cannot unload the venous system ; and the arteries by their own organic contractility force the blood into the swelling veins, and the balance of the circulation is completely broken. In the digestive organs, the functions languish, or are entirely suspended for a time, and, of course, the gastric, intestinal, and hepatic secretions are all deranged in quantity and quality.

The effects of *spirituous* and *malt* liquors on the human fabric are considerably different, as was observed, and humorously painted by Hogarth, in his caricatures entitled *Gin Lane and Beer Alley.* The *ale*-bibber is represented fat and unwieldy, as John Bull is generally drawn ; the *dram*-drinker is lean, haggard, furious in his countenance, with a dash of despair.

When we survey the infinity of substances which possess the *inebriating principle*, we are naturally led to ask what it is ? But this question is not so easily answered; for it would appear that it is not *one common element* diffused among all the class of intoxicating matters ; but that there are several different principles which have this peculiar effect. Chemistry has not yet cleared up this point. The Rhenish wines, for instance, which contain very little alcohol, are as *heady* as those of the South, which contain treble the quantity of spirit. Neither has it been quite ascertained in what manner the inebriating principle acts on the sensorium ; whether by absorption, or through the medium of the nerves. Although alcohol, we believe, has never been detected in the blood, yet there can be little doubt that the sanguineous and other fluids of the body are occasionally impregnated therewith in habitual drunkards, and in excessive debauches. It is said that the body of Alexander the Great was preserved a long time after death by the quantity of liquors which he drank during life. It is worthy of remark, that the more the urinary secretion is augmented, while swallowing liquors, the less inebriation is produced. This is recorded in an ancient proverb, which runs thus :—" *qui bene bibit, bene minxit.*" There is great reason to believe also that much of the intoxicating principle is carried off by perspiration; and in this way we can account for the sudden increase of drunkenness which takes place on going out into the cold air after full drinking. On account of the diuretic quality of Hollands and Geneva, it is more than probable that these liquors are, upon the whole, less deleterious to the constitution than rum, brandy, or whiskey. These reflections naturally lead us to the question, is there any anti-inebriating substance in nature? Plutarch informs us that Drusus, the son of Tiberius, always ate four or five bitter almonds before a feast, which enabled him to make all his companions drunk without being so himself. Aristotle, Hippocrates, and Galen consider a clove of garlic (probably from its diuretic power) as a preservative of the same kind. At this time, the bitter almonds, oil, and coffee are used on the continent as anti-inebriates. It is well known that plunging a drunken man into cold water has often the effect of bringing him to his senses, as is frequently seen when intoxicated sailors fall over-board; but the experiment is not devoid of danger, and is not to be recommended. There is a general opinion that an occasional or even a periodical debauch in drink is salutary. This has passed into a proverb in France———

> Qu'il faut, à chaque mois,

> S'enivrer au moins une fois.*

And it is not improbable that when the excess produces sickness and vomiting, (as it generally does among the unaccustomed to drink) the effects may be salutary. This we have frequently observed; but we are convinced that the succeeding good health and elasticity of spirits were principally owing to the vomiting; and that an emetic, without any intoxicating drink, would have had the same effects.

3. *Convulsive Drunkenness.* There is a species of ebriety attended with convulsions, which has not been sufficiently noticed in this country, but which must have fallen under the observation of almost every man.

All kinds of drink will produce this convulsive ebriety in particular, that is, in very irritable constitutions. But it is principally among soldiers and sailors, after drinking new rum, new wine, or adulterated spirits of any kind, especially if they are exposed to an ardent sun, that we meet this formidable complaint. It is for the most part, a few hours after the debauch that the convulsive symptoms appear. The man may leave the punch house, walk some way, and get home, without exhibiting any other than the common symptoms of intoxication; but presently he begins to feel a burning heat at the stomach; a giddiness in his head; a pain across the os-frontis, which induces him involuntarily, as it were, to press it with his hand. His eyes sparkle, and his countenance becomes haggard, with subsultus tendinum and stertorous breathing. To these symptoms are added nausea, followed by convulsions, in which, if the unfortunate wretch happen to be alone, he may dash himself against the walls or floor, or precipitate himself headlong into the street. In this way we have seen two men perish.

There is little doubt but that this orgasm in the nervous and muscular systems, together with the pain in the head, arise from the great irritation in the stomach, the centre of sympathies, whence is propagated, by a species of irradiation, the same irritation to other organs and parts.

The first thing to be done when called to a case of this kind is

* "A man should get drunk at least once a month."

to secure the patient, by sheets or napkins round the extremities and trunk, rather than by assistants; for it is not uncommon to see men seized with convulsive fits merely from the sight of another struggling in the same. Although the first and paramount indication is to evacuate the contents of the stomach, yet we should be cautious in exhibiting emetics. A small dose may prove inefficacious, and a large one may rupture the stomach, or increase the delirium and convulsions. Warm water, presented in a wooden or metal vessel, lest the patient should crash a glass one with his teeth, is the best remedy at the beginning. After each act of vomiting, which ought to be solicited with a feather, the patient revives a little, but soon falls back into the previous condition. If warm water and oil prove inefficient, we may add some oxymel of squills, but antimonial emetics are dangerous in convulsive drunkenness, of which we could adduce many proofs, but the following case will suffice as an example.

A dragoon having drunk nearly a quart of brandy, fell into the state above described, of convulsive ebriety. Being carried to an hospital, the physician, mistaking the nature of the complaint, and supposing the convulsions to arise from indigestible substances in the stomach, as often happens, ordered two grains of emetic tartar to be exhibited, and repeated thrice before it operated. During this interval, the delirium and convulsions arose to a frightful pitch. The spasms and contortions were so violent that it was almost impossible to hold him; and when confined, he struggled so furiously as to cause all his joints to crack—in fact, to threaten a general dislocation. The vomiting came on in paroxysms, and was excessively violent. After repeated alternations of fury and syncope, the patient became a little sensible; but this calm lasted only a short time. It was succeeded by such excruciating pains and cramps in the stomach and hypochondria that he uttered the most piercing cries. This again was followed by a vomiting of blood in considerable quantities. The patient spat blood for a long time afterwards, and was affected with an obstinate tremor in all his limbs, which required a protracted use of warm bathing to remove.

In eighteen cases of convulsive inebriation, we only administered an emetic to one, and we had great cause to repent of our proceeding. He was a young cavalry officer in the Regiment of Berry, who, after a hearty dinner, drank a bottle and a half of a very strong aromatic Flemish liqueur. After this excess, he went out to walk with some of his comrades in a garden, outside of the city, where he diverted himself in singing and dancing. Finally, he took it in his head to undress himself, though the weather was by no means warm, and this in spite of the remonstrances of his companions. He now tore even the shirt off his back, and his gaiety entirely changed to sullen sadness; which, in its turn, gave way to the most frightful and furious phrenzy. He threw himself on the ground, tore up the earth with his nails, gnawed the grass with his teeth, rolled about among bushes and thorns, and sent forth such terrific howlings as put the whole neighbourhood in a panic! Several of us ran to his assistance, and endeavoured to secure him with our cloaks and sashes, but he wounded every one of us before we could accomplish this object. After administering some tea and warm water, without producing vomiting, we ventured to exhibit two grains of emetic tartar, which dose was repeated in three quarters of an hour, founding the indication on the indigestion which might have resulted from the full meal taken immediately before the liqueur. The convulsions and phrenzy now became more violent than ever. He broke all the bands by which we had secured him to a tree, and we found it impossible to hold him down. Never was seen a more horrible spectacle; and what added to the distress was, that three officers who were present, caught, by sympathy, the complaint, and were seized on the spot with convulsions. Fortunately, however, a few pails of cold water poured on their heads, restored them to their senses and to tranquillity. By means of forcing down a quantity of warm water, vomiting was at length brought on, but it was not till midnight that the convulsions and cramps were conquered by means of sedatives, oily frictions, and opiate applications. These two cases may be sufficient to put us on our guard against the exhibition of antimonial emetics in convulsive drunkenness, especially where the inebriating material has been of a spirituous and fiery nature, or where there is any apprehension of inflammatory action resulting from violent spasm in the stomach.

In the year 1804, we were called to the assistance of a field officer attached to the Etat-Major of the sixth Corps, in the camp of Montreuil, remarkable for affability of manner, and gentleness of disposition. He had been engaged in a drinking match, where he had taken a considerable quantity of mulled wine, not wishing to be thought inferior to the other members of these Bacchanalian Orgies. At nine o'clock in the evening, not finding himself well, he returned to his apartments, where, his host and hostess having a large party, he behaved, to their utter astonishment, in the most scandalous manner. It was soon perceived that he was intoxicated, and he was solicited to retire to his chamber. He now conceived himself insulted, and threatened personal violence to any one who approached him. It was with the greatest difficulty that he was overpowered, disarmed, undressed, and confined to his bed by force. The servant presented him some weak tea, but he seized the vessel in which it was contained, and crashed it convulsively between his teeth into pieces. He seemed to applaud his finesse, in thus evading the drinking of the tea. He now uttered the most bitter invectives against us all, and it was with difficulty we could prevail upon him to swallow some drink. Two hours passed thus before vomiting ensued; but this was the signal for a calm after the tempest. He became reasonable, and submitted to our injunctions. An opiate afterwards procured sleep, and he awoke with only some lassitude and debility. He was, however, so ashamed of the affair, that he requested and obtained his dismissal. We afterwards met with him in a high official situation in a Foreign Court, but he persisted that he had never before seen us.

Ipecacuan, though not so dangerous an emetic, in these cases, is not to be had recourse to till warm water has been tried in vain. If the convulsions continue after the vomiting, venesection is necessary, and especially if the stomach be threatened with any inflammatory affection, or if fever arise.

Blood-letting is also necessary where vomiting cannot be induced, since the weakness which it occasions is favourable to nausea and vomiting. The warm bath is hurtful at the beginning; but after the stomach is disgorged of its contents, it is of great service, both in tranquillizing the system, preventing fever, and obviating those pains and that lassitude which follow convulsive inebriation. Open bowels and very light food, with diluent drinks, ought to be subsequently prescribed. The following case, which was communicated to us by Dr. Voisin, a distinguished practitioner of Versailles, will be read with interest.

" In 1810, a soldier addicted to drink, was charged with the care of conducting three young conscripts to St. Germains, where he lodged in the same chamber with them, on the second floor. Two of the conscripts retired early to bed, and the soldier, having got intoxicated, endeavoured, on his return, to force them from their bed, in order to occupy it himself. This they resisted, and thrust him out of the room. He made much to do at first, but presently lay down and fell asleep on the stairs. The third conscript, on returning home, found the drunken soldier under his feet, and on knocking at the chamber door, would be admitted only on condition of not bringing the drunken man in with him. During the night they heard him toss about violently on the ground; but as he had inspired them with more horror than pity, by his maltreatment of them, they uncharitably withheld from him all assistance. In the morning, this wretched soldier was found on the first flight of steps, covered with wounds and bruises, and quite dead! The conscripts were suspected of having committed murder —a coroner's inquest was called—surgeons examined the body, and attributed his death to the apparent wounds he had received. The conscripts were imprisoned.

Dr. Voisin having been consulted by the magistrates, conceived that the medical decision was premature, and though the body had been buried several days, it was disinterred, and Dr. V. in the presence of the magistrates and surgeons, proceeded to prove and substantiate the following positions:

" 1st. That the wounds were not in themselves mortal; that the vessels of the dura and pia mater were gorged with blood, as well as those of the plexus choroides; and that the ventricles of the brain contained a considerable quantity of water.

" 2d. That the inferior lobes of the lungs were also gorged with blood in a dissolved state; that the stomach was distended with gas, and contained near a pint of dark-looking flocculent liquid, exhaling a strong odour of brandy. The cardiac and pyloyric orifices of the stomach were inflamed, and the mucous membrane was speckled with red spots throughout its extent. From these pathological facts, Dr. Voisin drew the following conclusion:

"The man whom we have examined was evidently in a state, 1st. of common drunkenness, which afterwards became convulsive, when he precipitated himself from the upper to the next floor, dashing himself upon the steps and on the pavement, so as to occasion the wounds and bruises that appeared on the surface of the body. Nevertheless, the death of the man was owing rather to the inflamed state of the stomach and the apoplectic condition of the brain, than to the external lesions."

This able physician thus saved the lives of three men who had only the sin of imprudence and uncharitableness, but not the guilt of murder, to answer for.

---

### II.

### *On the Efficacy of* copious *local Bleeding in various Complaints characterized by Pain and Spasm, as well as Inflammation.* By J. F. Vaidy, M. D.

General blood-letting has often failed in general diseases, from the timid manner in which it was employed; and there is great reason to believe that local blood-letting, in local diseases, especially those termed *spasmodic*, has not been carried to an extent sufficient to insure success, in numerous instances. Our continental brethren employ this kind of evacuation much more frequently, and perhaps much more successfully than we do; therefore, we shall be wise in taking a lesson and a hint from our rivals——

Fas est et ab hoste doceri——

In the Journal Complimentaire des Sciences Medicales, for April 1819, M. Vaidy has related a great number of cases of various local affections, where very copious local bleeding, generally from thirty to forty leeches at a time, produced the most decisive and beneficial effects. We shall cursorily glance at some of these cases.

1. The *first* was a severe sciatica, which resisted general means. Three applications of thirty leeches each, completely removed the disease.

2. The *second* patient was M. Panckoucke, the Editor of the Dict. des Sciences Med. who was afflicted for some years with a most obstinate sciatica, that was not relieved by repeated applications of moxa, blisters, &c. In a recent and fierce attack, M. Vaidy was called, and applied twenty leeches to the thigh and ten to the leg. As the blood flowed the pain subsided. Next day, thirty-five leeches were necessary, and the relief was still greater than before. A third application of thirty leeches, a day or two afterwards, removed the disease; which, in previous attacks, had continued some months. Several other cases of this complaint are here stated, all tending to confirm the foregoing.

3. *Tooth-ache.* A lady was seized with a violent tooth-ache, and inflammatory swelling of the jaw. She had neither eat nor drunk for four days. Twelve leeches were applied to the angle of the lower jaw. She was instantly relieved, and fell into a profound sleep; on awaking, the complaint was gone.

4. *Gout.* Durand, a French Soldier, was received into the Val-de-Grace Hospital, with gout in several of the joints, but especially in the right knee, where the pain was excruciating. Thirty leeches were applied round the knee, and that night the patient had some sleep, to which he was a stranger for some nights previously. In a day or two the pain subsided, and with it the symptomatic fever. He returned to his duty. Two months afterwards, he had another attack, which was treated in the same way, and with similar success.

5. *Acute Rheumatism.* Vallet, a soldier, 24 years of age, was seized with acute rheumatism of the right arm and knee in particular, accompanied with intense fever, head-ache, and total loss of sleep. He was bled largely from the arm, which moderated the constitutional symptoms, but the local affection remained the same. Twenty-leeches were applied to each of the pained members, with almost instantaneous relief.

6. *Lumbago.* M. Vaidy employs copious local blood-letting in this complaint, followed by purgatives, and with the best success.

7. *Chronic Pleurisy.* Froisant, about 50 years of age, had experienced, for two years, a pungent pain in the left side of the chest, particularly under the shoulder blade, for which he tried several remedies. M. Vaidy ordered thirty leeches to be applied as near the spot as possible. The pain was removed at once, and did not afterwards return.

8. In the month of January, 1819, M. C. a musician had been, for some time, annoyed with a stitch in the left side, which checked his breath, and prevented him following his profession. M. Vaidy recommended him to abstain from wine, coffee, and *carminatives*, which he had been taking for his complaint, it being attributed, both by himself and his medical friends, to *wind*. The application of fifteen leeches to the side completely removed this "*windy stitch*," which all the carminatives he could swallow were unable to effect.

Here Dr. Vaidy exclaims against the routine custom of giving aromatics, stimulants, and carminatives, in what are termed *spasmodic* affections; but which, in reality, are chronic inflammations, that are kept up and rendered worse by the practice.

9. *Chronic Pulmonitis.* Deale, about 40 years of age, was for some time affected with a deep and fixed pain under the left nipple. This region of the chest, when examined by percussion, sounded badly. He was obliged to sit upright in bed. Thirty leeches were applied to the seat of pain, which was thereby greatly relieved, but the dyspnœa remained. A seton was cut in the side, and the wound bled so profusely, that the seton was obliged to be withdrawn. The day following, the patient was pale and depressed; but the respiration was free, and the pain in the side scarcely perceptible. The fright occasioned by the hæmorrhage was now converted into joy at the delivery from his complaints. This soldier returned in a few weeks to his military duties.

Various other cases, of a similar tendency to the foregoing, are related, all evincing the utility of carrying *local blood-letting* to a much greater extent than we are accustomed to do. We hope the attention of the faculty, on this side of the channel, may be strongly directed to this interesting point of therapeutics.

---

